# Overexpression of *chalcone isomerase A* gene in *Astragalus trigonus* for stimulating apigenin

**DOI:** 10.1038/s41598-021-03704-y

**Published:** 2021-12-17

**Authors:** Nagwa I. Elarabi, Abdelhadi A. Abdelhadi, Ahmed G. M. Sief-Eldein, Ismail A. Ismail, Naglaa A. Abdallah

**Affiliations:** 1 Department of Genetics, Faculty of Agriculture, Cairo University, Giza, 12613 Egypt; 2 Tissue Culture Unit, Ecology and Dry Land Agriculture Division, Desert Research Center (DRC), 11753 El-matarya, Cairo, Egypt; 3 Department of Biology, College of Science, Taif University, P.O. Box 11099, Taif, 21944 Saudi Arabia; 4 National Biotechnology Network of Expertise, Cairo, Egypt

**Keywords:** Biotechnology, Genetics

## Abstract

Apigenin is one of the most studied flavonoids and is widely distributed in the plant kingdom. Apigenin exerts important antioxidant, antibacterial, antifungal, antitumor activities, and anti-inflammatory effects in neurological or cardiovascular disease. *Chalcone isomerase A* (*chiA*) is an important enzyme of the flavonoid biosynthesis pathway. In order to enhance the apigenin production, the petunia *chi A* gene was transformed for *Astragalus trigonus*. Bialaphos survived plants were screened by PCR, dot blot hybridization and RT-PCR analysis. Also, jasmonic acid, salicylic acid, chitosan and yeast extract were tested to evaluate their capacity to work as elicitors for apigenin. Results showed that yeast extract was the best elicitor for induction of apigenin with an increase of 3.458 and 3.9 fold of the control for calli and cell suspension culture, respectively. Transformed cell suspension showed high apigenin content with a 20.17 fold increase compared to the control and 6.88 fold more than the yeast extract treatment. While, transformed T_1_ calli derived expressing *chiA* gene produced apigenin 4.2 fold more than the yeast extract treatment. It can be concluded that the highest accumulation of apigenin was obtained with *chiA* transgenic cell suspension system and it can be utilized to enhancement apigenin production in *Astragalus trigonus.*

## Introduction

Several plants develop naturally occurring polyphenols that play a remarkable role to protect against microbial infection^1^, antiallergic, anti-inflammatory, antitumor, antiviral, antioxidant and anticancer^2^. Flavonoids among phytochemicals types called polyphenols that were produced by widely plant species^3^. Flavonoids are considered plant secondary metabolites that have important physiological functions, pharmacological activities and are increasingly widespread because of their anticancer and antioxidant properties, low toxicity and multiple beneficial bioactivities^2,4,5^. Flavonoids are mostly distributed in, vegetables, fruits, seeds, stems, and flowers^6^. Flavonoids enhance the defense mechanism in plants against microbial infection and viruses^1^. Apigenin is among the most ubiquitous plant flavonoids, it possesses diverse pharmacological activities^7^. The anti-diabetic activity of apigenin could be attributed to its interaction with the cell reactive oxygen species (ROS)^8,9^, its ability to prevent glycosidase activity and increase secretion of insulin^10^.

*Astragalus L.* is the largest genus in the family Fabaceae (Leguminosae), which includes more than 2000 species disseminated mainly in the tropical African mountains and northern temperate regions, with 32 species domestic to Egypt^11^. Three main classes of important compounds (saponins, flavonoids, and polysaccharides) were extracted from *Astragalus* species^12^. Other compounds are possessing biological activity such as sesquiterpene-flavonolic complexes^13^, sterols, lignans, coumarins, and phenolic acids^14^. *Astragalus trigonus* has been utilized as a traditional medicine that improves the resistance to nephritis, heart tonic, hepatoprotective, diabetes and viral infections^15^. In China, *Astragalus* roots are utilized as traditional medicine, due to the anti-neoplastic, anti-diabetic and antioxidant properties of their compounds^16^. Roots of *Astragalus* species are used to treat leukemia and for wound healing in Turkish folk medicine^17^.

Elicitation has been utilized as an effective strategy to promote the biosynthesis of secondary metabolism compounds in plant cell cultures^18^. However, secondary metabolites production can be improved using the elicitor treatment of the undifferentiated cells including chitosan, salicylic acid and methyl jasmonate in many cases^19^.

In plant-cell, the phenylpropanoid pathway is the major pathway for flavonoids synthesis that includes three enzymes (cinnamate 4-hydroxylase (4CH), phenylalanine ammonia-lyase (PAL) and 4-coumaroyl CoA ligase (4CL))^20^. After that, the chalcone synthase (CHS) condenses 4-coumaroyl-CoA with three molecules of malonyl-CoA, producing naringenin chalcone, the main component for all flavonoids^20^. The heterocycle C closure is catalyzed by chalcone isomerase (CHI), which produced naringenin, the pre-product for eriodictyol (flavanone) and apigenin and luteolin (flavones). The flavone synthase (FNS) is required for apigenin production. Apigenin is then the substrate for the flavonoid 3′-hydroxylase (F3′H), giving rise to luteolin^21^.

Chalcone isomerase is one of the main enzymes in the flavonoid biosynthetic pathway that can convert chalcone to (2S)-naringenin. The heterocycle C closure is catalyzed by chalcone isomerase (chi), which generates naringenin, the precursor for apigenin, luteolin (flavones) and for eriodictyol (flavanone). The chalcone isomerase was cloned from *Ginkgo biloba* L^22^. In vitro enzyme activity, assayed by HPLC, indicated that recombinant *Gbchi* protein could catalyze the formation of naringenin from 6΄-hydroxychalcone. The expression of the *chi* activity showed positive correlation with the changes in transcription level of the *chi* gene, *Gbchi* activity was also positively correlated with total flavonoid levels in ginkgo leaves, suggesting that *chi* is a key gene in regulating flavonoid accumulation in ginkgo leaves^22^. Many plants were reported as sources for *chi* genes isolation including *Petunia hybrid*^22^, *Vicia narbonensis*^23^, *Zea mays*^24^, *Medicago sativa*^25^, *Phaseolus vulgaris*^26^, *Pisum sativum*^27^ and *Saussurea medusa*^28^. *Chalcone isomerase* genes were characterized in *Petunia*^23^*, Saussurea medusa*^28^ and *Boesenbergia rotunda*^29^*. Petunia chi* gene overexpression in tomato showed an increase about 78-fold in fruit peel flavonols, mainly due to rutin accumulation^30^. The objects of this study were to enhancement the production of the apigenin using different elicitors (jasmonic acid, salicylic acid, chitosan and yeast extract) and the overexpression of *chalcone isomerase A (chiA)* gene.

## Results

### *Agrobacterium* transformation of *A. trigonus*

**Figure 1:**
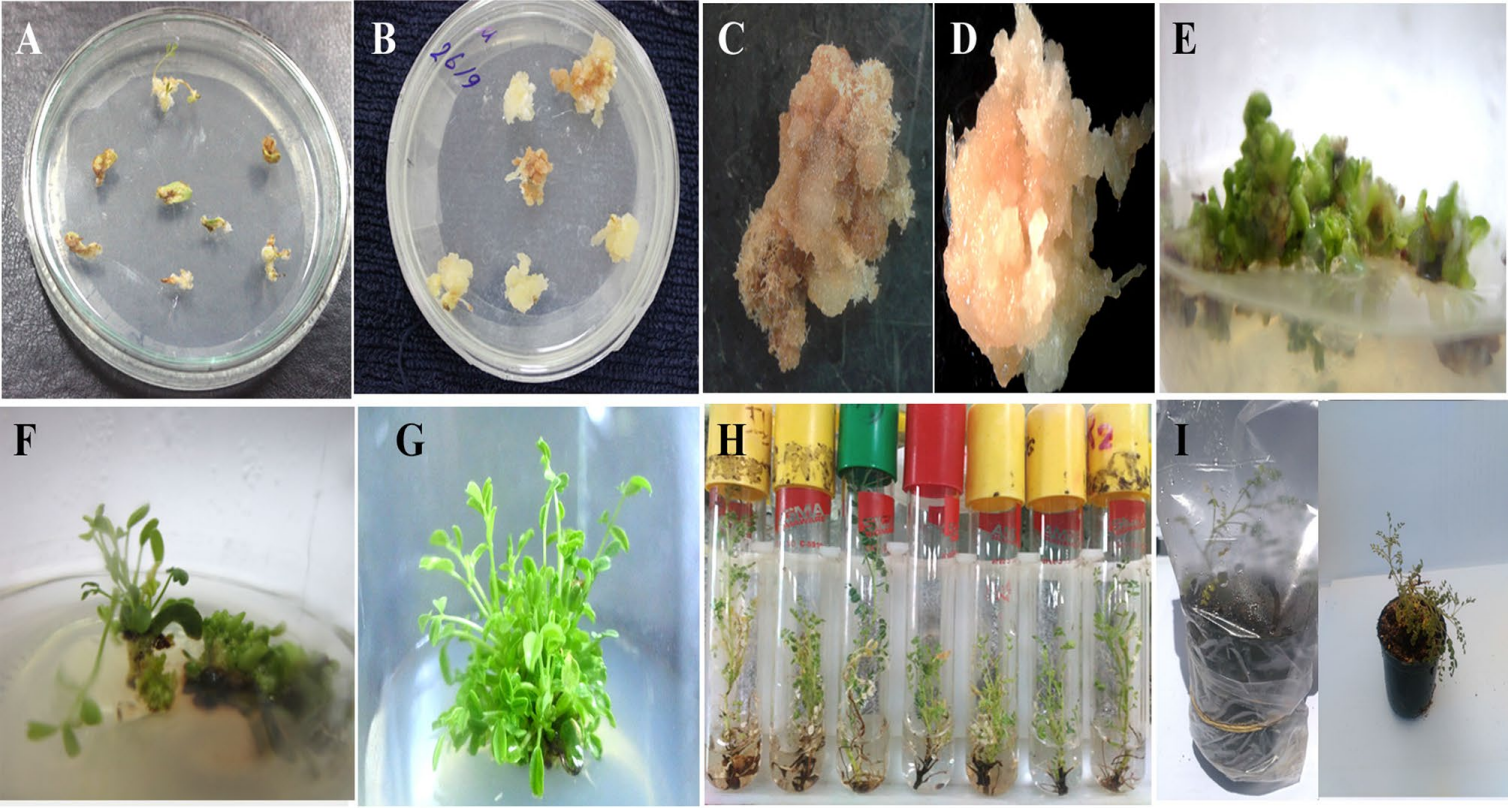
The regeneration stages of transformed *A. trigonus* plants. (**A**) The start explant; (**B**) Transformed calli on MS medium with bialaphos; (**C**) non-transformed callus on selective MS medium; (**D**) transformed callus on selective medium (survival calli); (**E**–**G**) shoot formation on shooting induction media after 10, 20 and 30 days, repressively; (**H**) Roots formation on rooting media and (**I**) Acclimatization of transformed *A. trigonus* plants (Acclimatization of transformed *A. trigonus* plants in mixture of mixture of sand and peat moss (1:1 v/v)).

The calli of *A. trigonus* were transformed using *Agrobacterium tumefaciens* harboring the binary vector pFGCS-*chiA*. Transformed calli were cultured into fresh MS media with 0.5 mg/l 2,4-D and 1.5 g/l bialaphos to develop and select transformed cells. The transformed calli were transferring within one month into the shoot induction media. Putative transformed shoots survive in the media containing 1.5 g/l bialaphos appeared within 10–15 days. Shoot initiation started after about 10 days from culturing and the multiple shooting formation started after 20 days from subculturing. Survived shoots were used for shoot elongation, root regeneration and acclimatization (Fig. 1).

### Screening of transgenic plants

The presence and integration of the *chiA* gene into the *A. trigonus* genome were confirmed in the DNA of all rooted plants by PCR and dot-blot hybridization analysis. Primers specific for the *chiA* gene were designed to produce a 484 bp core fragment of the integrated gene. PCR analysis characterized three out of six bialaphos resistant plants, as having the same PCR band pattern resulting from the positive control (Fig. S1).

Also, DNA from the six bialaphos resistant plants was subjected to dot blot hybridization analysis for further confirmation of the insertion of the *chiA* gene in the *A. trigonus* genome. Isolated DNA was blotted and hybridized with the *chiA* probe. Results from the dot-blot assay gave positive hybridization results with the same three PCR positive plants, confirming the integration of the transgene in the genome (Fig. S3).

PCR and dot blot positive lines were further analyzed for studying the expression of the transgene using RT-PCR and northern hybridization. Total RNA was isolated from six bialaphos resistant plants as well as non-transformed plant transformed (negative control). RT-PCR assay was carried out using isolated RNA to amplify the cDNA from positive expressed transgene to test the expression of *chiA* mRNA in six transformed and non-transformed plants. The ORF-specific *chiA* primers successfully amplified fragments to the expected 726 bp fragment in the three positive transgenic plants. No band was detected in the negative control (Fig. S2A). RT-PCR results confirm the positive transcription of the full gene in three transgenic lines.

Northern blot analysis was carried out using isolated RNA from the six putative transformed plants as well as the control to detect transcripts that hybridized specifically the probe derived from the *chiA* gene. The northern analysis indicated that a clear band specifically hybridized with the probe prepared from the *chiA* gene of the three transformed plants but was not detected in the other three lines and the non-transgenic plants (Fig. S2B).

### Elicitation of apigenin production

*A. trigonus* calli and cell suspension were incubated with different concentrations (50, 100, 150 and 200 µM) of each of jasmonic acid and salicylic acid while (50, 100, 150 and 200 mg/l) from yeast extract and chitosan. Accumulation of apigenin in response to different concentrations of elicitors was evaluated using HPLC before and 6 weeks after treatments. Calli and cell suspension without any treatment of the elicitors are considered as control.

Among the four elicitors used, YE, CH and JA have been more effective in increasing the apigenin accumulation compared to the control in both calli and cell suspension cultures. For calli culture, treatment of YE at 100 mg/l was exhibited maximum accumulation (2.786 mg/g), which corresponds to an increase of 3.48 fold as compared to the control (0.801 mg/g) (Table 1). In response to different levels of CH used, a concentration of CH at 50 mg/l was found to be effective with maximum apigenin accumulation (1.951 mg/g), which corresponds to an increase of 2.43 fold as compared to the control. Different levels of JA used showed that 200 μM JA was the effective concentration for apigenin accumulation 1.621 mg/g, with an increase of 2.02 fold compared to the control. The calli weight showed slides increase or decrease due to elicitor treatment except for JA and CH. The highest percentage of increase in fresh weight was obtained with 200 µM jasmonic acid, with an increase of fold 3.66 compared to weight before elicitor treatment. In general, results revealed that there is no clear correlation between increasing the concentration of the elicitor and the apigenin contents and the increase of calli weight. In addition for cell suspension culture, treatment of YE at 100 mg/l also was showed maximum accumulation (3.793 mg/g), which corresponds to an increase of 3.99 fold as compared to the control (0.951 mg/g). In response to different levels of CH and JA used, a concentration at 100 mg/l of CH and JA in the cell suspension were found to be effective with maximum apigenin accumulation (2.048 and 2.047 mg/g respectively), which corresponds to an increase of 2.15 fold as compared to the control (Table 1).

**Table 1:** Effect of elicitor treatments on apigenin contents.

Elicitors types	Elicitors concentrations	*A. trigonus* Calli		*A. trigonus* Cell suspension	
		Mean apigenin content (mg/g)	Apigenin change (fold) compared with the control	Mean apigenin content (mg/g)	Apigenin change (fold) compared with the control
Control (MS free)	0	0.801 ± 0.04	1.00	0.951 ± 0.060	1.00
SA (µM)	50	0.769 ± 0.030	0.96	1.361 ± 0.300	1.43
	100	0.897 ± 0.060	1.12	1.658 ± 0.200	1.74
	150	0.467 ± 0.040	0.58	0.965 ± 0.040	1.01
	200	0.384 ± 0.009	0.48	0.364 ± 0.030	0.38
YE (mg/l)	50	1.951 ± 0.090	2.43	2.198 ± 0.100	2.31
	100	2.786 ± 0.400	3.48	3.793 ± 0.100	3.99
	150	1.724 ± 0.300	2.15	2.483 ± 0.300	2.61
	200	1.396 ± 0.300	1.74	1.978 ± 0.100	2.08
CH (mg/l)	50	1.860 ± 0.090	2.32	0.325 ± 0.070	0.34
	100	0.191 ± 0.020	0.24	2.048 ± 0.300	2.15
	150	0.101 ± 0.020	0.13	0.248 ± 0.080	0.26
	200	0.654 ± 0.120	0.82	0.785 ± 0.120	0.83
JA (µM)	50	0.185 ± 0.040	0.23	0.245 ± 0.020	0.26
	100	1.416 ± 0.150	1.77	2.047 ± 0.080	2.15
	150	0.921 ± 0.040	1.15	2.102 ± 0.120	2.21
	200	1.621 ± 0.100	2.02	1.782 ± 0.100	1.87

HPLC was used for measuring the apigenin concentration in the six weeks *A. trigonus* calli and cell suspension cultures incubated on MS media supplemented with different concentrations of SA, YE, JA and CH.

### Accumulation of apigenin in transgenic *A. trigonus* calli and cell suspension

**Figure 2:**
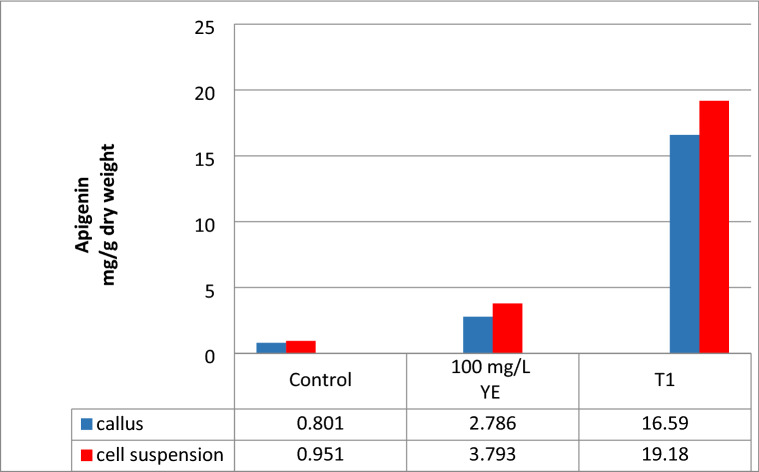
Apigenin contents in calli and cell suspension of the T_1_ *chiA*-transgenic and the nontransgenic (control) *A. trigonus* measured by HPLC.

Calli and cell suspension culture regenerated from T_1_ *A. trigonus* plants harboring the *chiA* gene were used for measuring apigenin contents using HPLC analysis. Untransformed calli and cell suspension were used as control. Apigenin was extracted from transformed and non-transformed tissue (calli and cell suspension) and its accumulation was measured by HPLC (Fig. S3). Each sample analysis consisted of three replicate measurements of the amount of each analytic. Transformed cell suspension exhibit high accumulated apigenin (19.18 mg/g dry weight) with a 20.17 fold increase compared to the control while the apigenin content was 16.59 mg/g dry weight with about 20.71 fold for transgenic calli. Comparison between the YE treatment (100 mg/g) and the *chiA* transgenic cell suspension showed that 5.05 and 5.95 folds increase for callus and cell suspension, respectively (Fig. 2).

## Discussion

*Astragalus* is the largest genus in the Fabaceae family with more than 2500 species of over 3000 species of herbs and small shrubs. Several flavanones were isolated from different *Astragalus* species. The ability to cultivate plant callus cells has made a significant contribution to plant biotechnology concerning the production of commercially beneficial compounds^31^. The good reproducibility and the high rate of cell growth make callus cultured suitable for the high-value secondary metabolites production and other important plant components^32^. Plant calli and cell suspension culture technology have been an effective system for commercially important natural products production^33^, including triterpene^34^, terpenoid^35^, phenylpropanoid^36^ and flavonoid^37^. In addition, cell suspensions have been involved in medicinal compounds production utilized as food additives and flavors, and coloring agents^38^. Many significant medicinal plants have been utilized for the production of important antiviral medicinal metabolites through callus and cell cultures^39^.

Apigenin is biosynthetically derived from the general flavone synthesis pathway and phenylpropanoid pathway^40^. There are many ways to improve the production of apigenin such as using elicitation or by enhancing gene expression using gene transfer methods. In this study, four biotic elicitors, JA, SA, CH and YE were used to improve apigenin production in *A. trigonus* calli and cell suspension. The results of this study indicated that 100 mg/l yeast extract scored the high apigenin content in both calli and cell suspension (2.786, 3.793 mg/g dry weight about 3.48, 3.99-fold of apigenin respectively). The data showed that the cell suspension was more effective in apigenin production than calli culture due to more exposed and absorption of cells than calli to the elicitors. Presently, yeast extract is usually used as an abiotic elicitor for the enhancement and induction of plant secondary metabolites production. George et al.^41^ reported that yeast extract was utilized as an elicitor to enhance plant growth, due to its high amino acid content. Previously, yeast extracts were utilized as growth nutrients such as callus cultures and crown-gall tissue cultures^42^. In vitro culture elicitation is a valuable method for extending and promote the production of desirable secondary metabolisms^43^. Recently, elicitors are considered as signal molecules that stimulate the signal-transduction pathways which lead to the expression and activation of some genes that are related to the biosynthesis of the secondary metabolites^44^.

Chalcone isomerase is an important enzyme of the flavonoid biosynthesis pathway by converting the isomerization of chalcone to flavanone^45^. Secondary metabolite production is commonly related to rapid, transient increases in enzyme activities of the phenylpropanoid/flavonoid pathway including flavonoid 3′-hydroxylase (F3′H)^46^ and chalcone isomerase A (chiA)^47^. In this research, the *chiA* gene was transformed into *A. trigonus* genome using *Agrobacterium* transformation. The results showed an increase of ~ 20 fold in apigenin production in transgenic T_1_ cell suspension and transgenic T_1_ calli, respectively compared to the control and ~ fivefold increase compared to treatment with 100 mg/l yeast extract. That indicated that over expression of *chiA* gene results in increasing the flavonoids including the apigenin. Various genes in the flavonoid pathway show differences in substrate specificity or preference in several plant species. *chiA* cDNA was first isolated from *Vicia narbonensis*^23^ and then from *Petunia* hybrid^22^. The overexpressing Peony *chiA* gene in transgenic tobacco led to an increasing in flavonols and flavones content^48^. In addition, the expression of onion *chiA* in tomatoes produced transgenic fruits with about 400 and 260 fold increased levels of anthocyanins^49^. These findings reveal the regulatory effects of *chi* genes on flavonoid and suggest that it is possible to obtain desirable agronomic traits through manipulating this enzyme. Our results showed that the transgenic *A. trigonus* expressing *chiA* gene showed increasing in apigenin content that due to the effect of chalcone isomerase in the flavonoid biosynthesis pathway. In general, we found that cell suspension culture is more efficient in production bio-products than calli.

## Conclusion

Elicitation is a promising strategy to over-produce flavonoids in plants. In this study, we provided an effective method for flavonoids apigenin production in *A. trigonus* callus and cell suspension culture. The impact of elicitation on biomass production of flavonoids in the callus and cell suspension culture was analyzed. The results depict the importance of cell suspension and callus for standardizing the best elicitor. The results showed enhanced accumulation of apigenin in the cell suspension and callus of *A. trigonus* when treated with yeast extract, jasmonic acid, chitosan and salicylic acid. Comparing data obtained from the HPLC showed that most elicitors witnessed the positive expression of apigenin at specific concentrations except for salicylic acid. The best elicitor was obtained with 100 mg/l YE. In addition, cell suspension and calli developed from a T_1_ transgenic plant expressing the *chiA* gene has magnified positive effects compared to all tested elicitors. Therefore, we recommend using transgenic plants for enhancing apigenin contents in *A. trigonus*. We concluded that transgenic *chiA* cell suspension culture is effective in enhancing the in vitro production of industrially important flavonoids. This is the first report about the effects of the *chiA* overexpression in *A. trigonus* for enhancing apigenin production.

## Materials and methods

### Plant material

*Astragalus trigonus* seeds were collected from a wild population from Northern West Coast; Ras El-Hekma, Matrouh, Egypt (31°06′59.2"N 27°46′42.7"E). The plant was classified by the taxonomist of the herbarium of Desert Research Center, Egypt according to Xu^50^. The plants were identified by Dr. Ahmed Gamal, Tissue culture unit, Ecology and Dry land Agriculture division, Desert Research Center (part of the research team), according to Flora of Egypt Cheklist^51^. Seeds were indexed to “Scientific Names of Flora of Egypt” including their families, habits, localities and abundances under its scientific name *Astragalus trigonus* DC. The collected plants included in the manuscript were deposited at The Herbarium of the Desert Research Center.

All experimental research complied with the Ministry of Agriculture and Land Reclamation bulletin with the most important technical recommendations. Permissions for using the seeds and conducting the experiments were obtained from the Desert Research Center.

### *Agrobacterium* transformation

**Figure 3:**
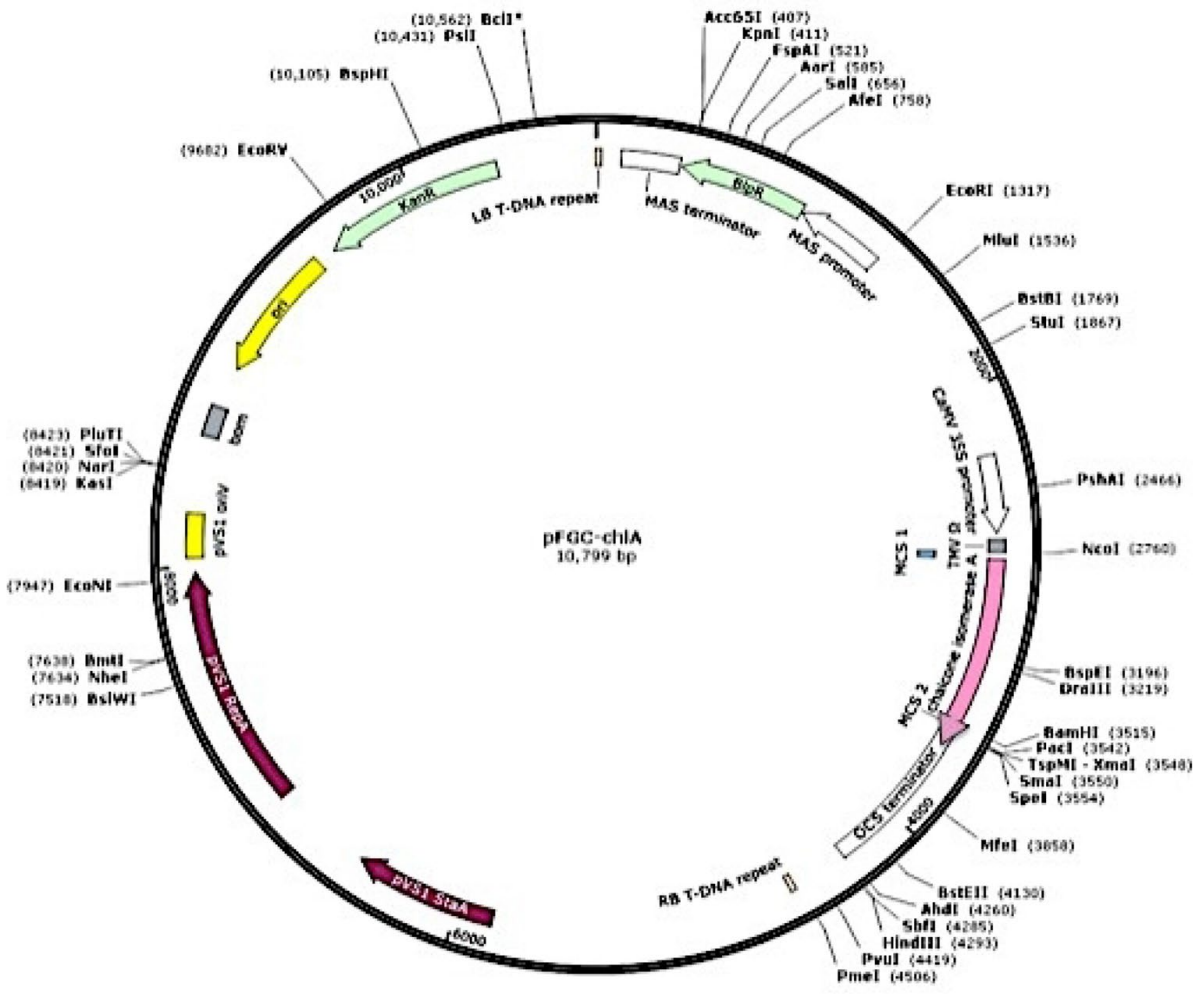
The plant vector pFGCS-*chiA* with *chiA* gene ORF (726 bp).

*Agrobacterium tumefaciens* strain LBA4404 having the plant vector pFGCS-*chiA* with petunia *chiA* gene (accession no. AF233637.1) was kindly provided from Dr. Naglaa A. Abdallah, Cairo University, Egypt and was used in transformation protocol (Fig. 3). *A. trigonus* calli explants were immersed in the *Agrobacterium* suspension for 5 min. Thereafter, the explants were blotted on a sterilized filter paper, placed onto a co-cultivation media (MS medium supplemented with 0.5 mg/l 2, 4-D) and were incubated under dark conditions^52^. After co-cultivation for three days, the transformed calli were transferred to MS media including 0.5 mg/l NAA, 0.5 mg/l BAP, 500 mg/l cefotaxime and 1.5 mg/l bialaphos (Sigma-Aldrich, Japan) to select the transformed calli for shoot induction^53^. Elongated shoots were successfully rooted in MS media supplemented with 1.0 mg/l NAA^15^, and incubated at 25 °C under 16/8-h light/dark photoperiodic regime (1000-Lux).

### Regeneration and cell suspension culture

The seeds were sterilized for 20 min using 0.5% (v/v) sodium hypochlorite (NaOCl), and then washing four times using dH_2_O. MS media^54^ were utilized for seed germination. Four-week-old in vitro hypocotyls were cultured on MS media supplemented with 0.5 mg/l 2,4-D for callus induction. For suspension cultures initiation, 1.25 g portions of friable *A. trigonus* calli (taken on the 25th day of the growth cycle) were inoculated into 125-ml Erlenmeyer flasks containing 50.0 ml of MS liquid medium enriched with 0.5 mg/l 2,4-D and 1.0 mg/l naphthaleneacetic acid (NAA) and agitated on a shaking shaker (120 rpm, 25 ± 2 °C, in dark). During that period, many small- and big-aggregates, clusters of cells and single cell are produced from the callus into the suspension. To separate the cell-suspension culture, fresh media was utilized for diluted the mother suspension cultures in ratio 1:1. This was performed using sterile meshes (0.5 mm) to collect the cell-aggregates and single cells. The cell-suspension culture was maintained by subculturing every 3 weeks on fresh liquid media and incubating under shaking conditions. Each experiment was repeated three times.

### Polymerase chain reaction (PCR)

*A. trigonus* DNA was isolated from the transformed leaves by the CTAB method according to Rogers and Bendich^55^. The reaction was performed using designed primers Chi-core-F 5′-CCCCAGGAGTTGACTGACTC-3′ and Chi-core-R 5′-ATTTCTCGGTCTCCGGTTTT-3′ to amplify a core region of 484 bp specific for *chiA* gene detection. The reaction mixture (20 µl) contained 10 ng DNA, 200 µM dNTPs, 0.5 units of Red Hot *Taq* polymerase (ABgene Housse, UK), 10-X *Taq* polymerase buffer, 2.5 mM MgCl_2_ and 1 µM of each primer. Samples were subjected to 94 °C for 5 min then 1 min at 94 °C; 1 min at 56 °C and 1 min at 72 °C for 35 cycles. Samples were separated using agarose gel electrophoresis (1.5%) and visualized with ethidium bromide and photographed.

### Dot blot analysis

DNA extracted from both putative transformed *A. trigonus* and non-transformed plants were blotted onto Hybond NC nylon membrane (Amersham, cat. # 7404-009, USA). Prehybridization and hybridization conditions were carried out as provided by the manufacturer’s recommendations^56^. The PCR product from *chiA* gene amplification was utilized as a probe. Biotin Chromogenic Detection kit (Ferments Life Sciences, cat. #K0661, cat. #K0662, USA) was utilized for hybridization and detection as described by the supplier’s instructions.

### Reverse Transcription PCR (RT-PCR)

SV Total RNA Isolation System (Promega, cat. # Z3100, USA) was utilized for total RNA extraction from both PCR positive plants leaves and non-transformed plants as control. Then RevertAid™ First Strand cDNA Synthesis Kit (Ferments Life Sciences, cat. # K1621, USA) was used to perform RT-PCR reaction with the designed Chi-core primers. The analyses were carried out on both transformed (PCR positive) and non-transformed plants (control) using the *chiA* specific primers and the RT-PCR products were visualized on 1.5% agarose gels.

### Northern bolt analysis

The extracted RNA was used in northern hybridization analysis. The hybridization and detection were performed using the Biotin Chromogenic Detection Kit as described by the supplier’s instructions (Ferments Life Sciences, cat. #K0661, cat. #K0662, USA). The PCR product resulting from *chiA* gene amplification was used as a probe. Primers were designed using primer3 software and used for amplifying the full ORF has the following sequences; chiA-F 3′-ATGTCTCCTCCAGTGTCCGT-5′ and chiA-R 3′-CTAGACTCCAATCACTGGAATAG-5′.

### Elicitation treatment

The calli and cell suspension of *A. trigonus* were cultured on MS medium. For elicitation experiment, concentrations of 50, 100, 150 and 200 μM were added to culture media for jasmonic acid (JA) and salicylic acid (SA), while 50, 100, 150 and 200 mg/l were added for chitosan (CH) and yeast extract (YE) treatments. The culture without elicitors was used as a control. Calli and cell suspension were cultured twice every three weeks on MS media supplemented with 0.5 mg/l 2,4-D and augmented with the elicitors^57,58^. Six weeks post-treatments, apigenin content (mg/g dry weight) were recorded for different treatments and compared to the control.

### Measurement of Apigenin

Apigenin ≥ 95.0% (HPLC) purchased from Sigma-Aldrich (cat. # 520-36-5) was used as standard for HPLC measurements. Standard stock solution of apigenin was prepared in methanol at 1000 μg/mL concentrations. An appropriate amount of the standard stock solution was mixed and diluted with methanol to obtain final concentration 10 μg/ml of the working standard solution for constructing the relevant calibration curves. The apigenin was extracted from elicitor treated calli, cell suspension, T_1_ transgenic calli and cell suspension expressing the *chiA* gene as well as the control. All samples were finely powdered and extracted with 10 ml of 80% methanol for 24 h at room temperature. The extracts were filtered through Whatman no. 1 filter paper, kept in the vacuum desiccator for seven days and the residue was then dissolved in 1 ml of methanol and filtered through 0.22 µM membrane filters^59^. Apigenin content was determined in the samples by Dionex UltMate 3000 HPLC system equipped with quaternary pump LPG3400SD, a WPS 3000 SL analytical autosampler, and a DAD-3000 photodiode array detectors (Thermo Dionex, Germany). Samples were run on an analytical column C18 using gradient elution. The mobile phase was a mixture of acetic acid: acetonitrile: phosphoric acid: methanol and H_2_O (10:100:100:200:200) at a flow rate of 0.6 ml/min and the column temperature was maintained at 30˚C. The quantification of apigenin was based on a standard comparison of 352 nm, the maximum absorbance of apigenin. The injection volume of the standard and samples solutions were about 6 µl and the chromatography system was equilibrated by the mobile phase. Data were analyzed and integrated by Chromeleon 7 software (Chromeleon™ Chromatography Data System (CDS) Software, Thermo fisher scientific, cat # CHROMELEON7, USA). Compound peaks were identified by comparing of the retention times and the ultraviolet–visible spectra of the samples with those of standard compounds. The data obtained by integration of the peaks for known amounts of standard were compared with the peak areas of the sample compounds for quantification.

### Ethical approval

Experiment was conducted after taking proper approval from the Faculty of Agriculture, Cairo University Committee, Egypt. All experimental research complied with the Ministry of Agriculture and Land Reclamation bulletin with the most important technical recommendations.

## Supplementary Information

Supplementary Figures.
