# Supplementary material for: Overexpression of chalcone isomerase A gene in Astragalus trigonus for stimulating apigenin
Source: Sci Rep. 2021 Dec 17;11:24176. doi: 10.1038/s41598-021-03704-y (PMC8683443; doi:10.1038/s41598-021-03704-y)
Supplement: Supplementary file 1 — Supplementary Figures. [file 41598_2021_3704_MOESM1_ESM.pdf]

Figure S1

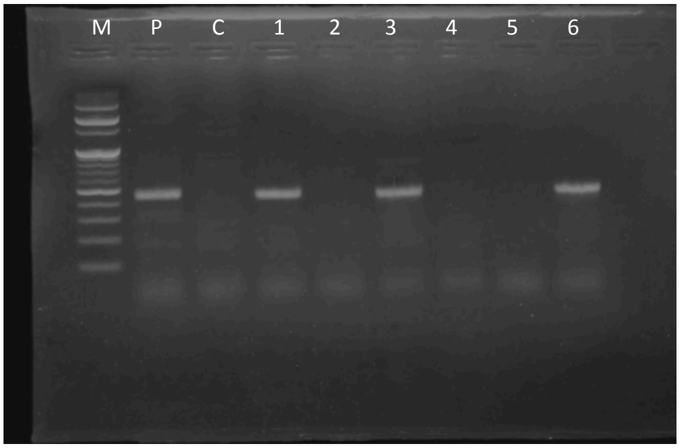

Molecular confirmation of *chiA* gene into transformed *A. trigonus* genome. PCR analysis confirming the transformation of the *chiA* gene into the plant genome. P: positive control (plasmid), C: negative control and lanes 1-6: transformed lines.

Figure S2

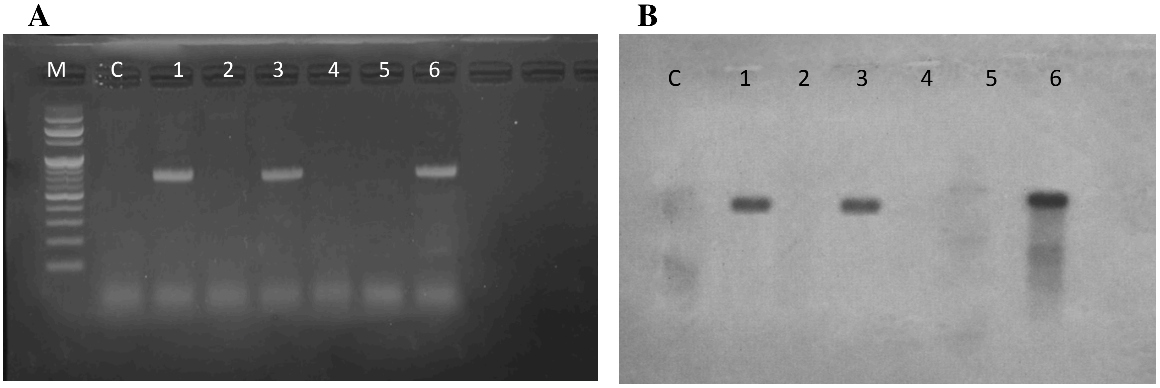

Analysis of *chiA* gene expression into transgenic *A. trigonus* plants using total RNA extracted from both the transformed lines and the control. (A) RT- PCR analysis of the *chiA* gene of transformed *A. trigonus*. M1: 100 bp DNA ladder, C: negative control; 1-6 putative transformed lines. (B) Northern blot analysis confirming the expression of the full *chiA* gene in three out of six putative transgenic lines (1-6) of *A. trigonus*. C: non-transgenic as a negative control.

Figure S3.

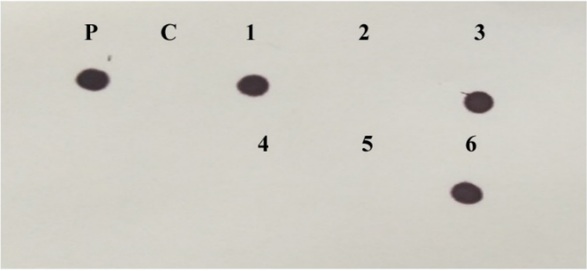

The dot plot analysis; P: positive control; C: negative control; from 1-6: six transgenic lines

Figure S3.

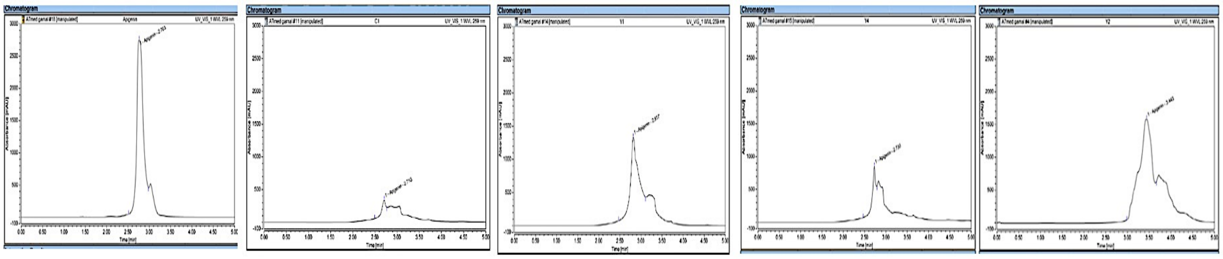

Apigenein standered | **Control calli** | **Transgenic T_1 calli** | **Control cell suspension** | **Transgenic T_1 cell suspension**

Representative HPLC chromatograms of the apigenin for the standard injection, calli and cell suspension extracts of transgenic and non transgenic *A. trigonus*

.
